# Unveiling the role of host kinases at different steps of influenza A virus life cycle

**DOI:** 10.1128/jvi.01192-23

**Published:** 2024-01-04

**Authors:** Soumik Dey, Arindam Mondal

**Affiliations:** 1 School of Bioscience, Indian Institute of Technology Kharagpur, Kharagpur, India; Indiana University Bloomington, Bloomington, Indiana, USA

**Keywords:** influenza, host kinases, MAP kinase, ERK, PKC, RNP assembly, viral transcription and replication, RNP export

## Abstract

Influenza viruses remain a major public health concern causing contagious respiratory illnesses that result in around 290,000–650,000 global deaths every year. Their ability to constantly evolve through antigenic shifts and drifts leads to the emergence of newer strains and resistance to existing drugs and vaccines. To combat this, there is a critical need for novel antiviral drugs through the introduction of host-targeted therapeutics. Influenza viruses encode only 14 gene products that get extensively modified through phosphorylation by a diverse array of host kinases. Reversible phosphorylation at serine, threonine, or tyrosine residues dynamically regulates the structure, function, and subcellular localization of viral proteins at different stages of their life cycle. In addition, kinases influence a plethora of signaling pathways that also regulate virus propagation by modulating the host cell environment thus establishing a critical virus-host relationship that is indispensable for executing successful infection. This dependence on host kinases opens up exciting possibilities for developing kinase inhibitors as next-generation anti-influenza therapy. To fully capitalize on this potential, extensive mapping of the influenza virus-host kinase interaction network is essential. The key focus of this review is to outline the molecular mechanisms by which host kinases regulate different steps of the influenza A virus life cycle, starting from attachment-entry to assembly-budding. By assessing the contributions of different host kinases and their specific phosphorylation events during the virus life cycle, we aim to develop a holistic overview of the virus-host kinase interaction network that may shed light on potential targets for novel antiviral interventions.

## INTRODUCTION

Post-translational modifications (PTM) are considered as one of the primary mechanisms for expanding the coding capacity and hence the proteome size of an organism (1, 2). In humans, extensive diversification of individual proteins through phosphorylation, ubiquitination, methylation, acetylation, glycosylation, poly-ADP ribosylation, etc. leads to at least one log increase in the number of protein species in comparison to the corresponding transcripts (2). This phenomenon is efficiently exploited by human viruses, specifically RNA viruses, which usually encode a limited number of gene products. The PTM that has been most extensively characterized for RNA viruses is phosphorylation where kinases covalently link a phosphate moiety to the hydroxyl group of the serine, threonine, tyrosine, or histidine residues present within the viral proteins (3–6). RNA virus genome does not encode for any known kinase, which means that they heavily rely upon host kinases for the phosphorylation of viral proteins, thereby establishing a virus-host relationship that is indispensable for their propagation (7).

Kinases are a group of proteins that participate in almost all cellular signaling processes through the phosphorylation of other proteins, lipids, or carbohydrates (3). The human kinome is constituted of 538 kinases (identified so far) that are categorized into different types including serine/threonine kinase, receptor tyrosine kinase (RTK) and non-RTK, histidine kinase, dual specificity kinase, and lipid kinase (8, 9). Phosphorylation at specific sites is required for structural transformation, functional activation, molecular interaction, and subcellular localization of a multitude of cellular biomolecules. Because of the importance of these phosphorylation events, small molecule kinase inhibitors have been recognized as anticancer or antiviral drugs (10). Kinase inhibitors can block specific signaling pathways and inhibit enzymes involved in the cell or virus growth and proliferation. Currently, most of the antiviral drugs are targeted toward viral proteins, which frequently suffer resistance from the newer virus strains generated through acquired mutations in the viral genome. Kinase inhibitors could be a better replacement of such traditional antiviral drugs as it is difficult for the virus to overcome the host dependency. Despite advantages, kinase inhibitors suffer off-target effects and associated toxicity that needs to be minimized for better efficacy as drugs (9). As of now, 72 small molecule protein kinase inhibitors have been approved as drugs by the U.S. Food and Drug Administration (FDA), among which 62 are against neoplasms (10). Unfortunately, no small molecule kinase inhibitor has been approved as an antiviral till date (10). A small molecule inhibitor of the mitogen-activated protein kinase (MAPK) kinase (MEK) is currently under phase I clinical trial (NCT04385420) as an anti-infective drug (11–13). Notably, it has completed a phase II study (NCT04776044) tested in COVID-19 patients (13). This highlights the knowledge gap that persists in terms of clear understanding of the role of host kinases in modulating the virus replication cycle, which is critical for exploiting them as potential antiviral targets.

Influenza viruses cause respiratory infection leading to seasonal epidemics (influenza A and B) and sporadic pandemics (influenza A) (14). Seasonal influenza outbreaks cause an estimated 650,000 deaths worldwide each year, where people aged 65 years or older, newborns, young children, and immunocompromised individuals are designated as the high-risk cohort (15, 16). The mortality rate could also be high among young adults (20–40 years) as observed during the 1918 pandemic (17, 18). This variability in pathogenicity along with differential transmissibility and host tropism is the signature of influenza A viruses due to their incessant course of evolution (14). The avian influenza viruses (IAVs or influenza A) naturally reside in wild aquatic birds (14). IAVs harbor 16 different hemagglutinin (HA) and nine different neuraminidases (NAs), which in different combinations decorate the outer envelope surface and define different subtypes, for example, H1N1, H3N2, and H5N1 (19). Owing to the segmented nature of the genome, two different IAVs can undergo genetic reassortment upon co-infection in a single host, thereby leading to the generation of newer virus subtypes through the process known as “antigenic shift” (20). Antigenic shifts are the major cause of emerging zoonotic outbreaks leading to sporadic epidemic and pandemic events. Additionally, mutations in the viral genome can change the antigenic property of HA and NA and thereby generate novel viral strains through “antigenic drift” (21, 22). These strains can evade pre-existing immunity and result in the recurrent seasonal outbreaks. The antigenic drifts and shifts together contribute to the evolutionary dynamics of influenza virus and also result in antiviral resistance. Currently, there are three classes of anti-influenza drugs: oseltamivir, targeting viral NA; adamantanes, targeting viral ion channel M2; and baloxavir marboxil, the polymerase acidic (PA) endonuclease inhibitor (23). Unfortunately, all of the seasonal strains of influenza A and B viruses show resistance toward adamantanes thus leaving oseltamivir and baloxavir marboxil as the two options to treat influenza virus illness (23). Clearly, there is a pressing need to develop novel therapeutic strategies, preferably by targeting the host rather than the viral proteins to outcompete the rapid evolution of the virus

**Fig 1:**
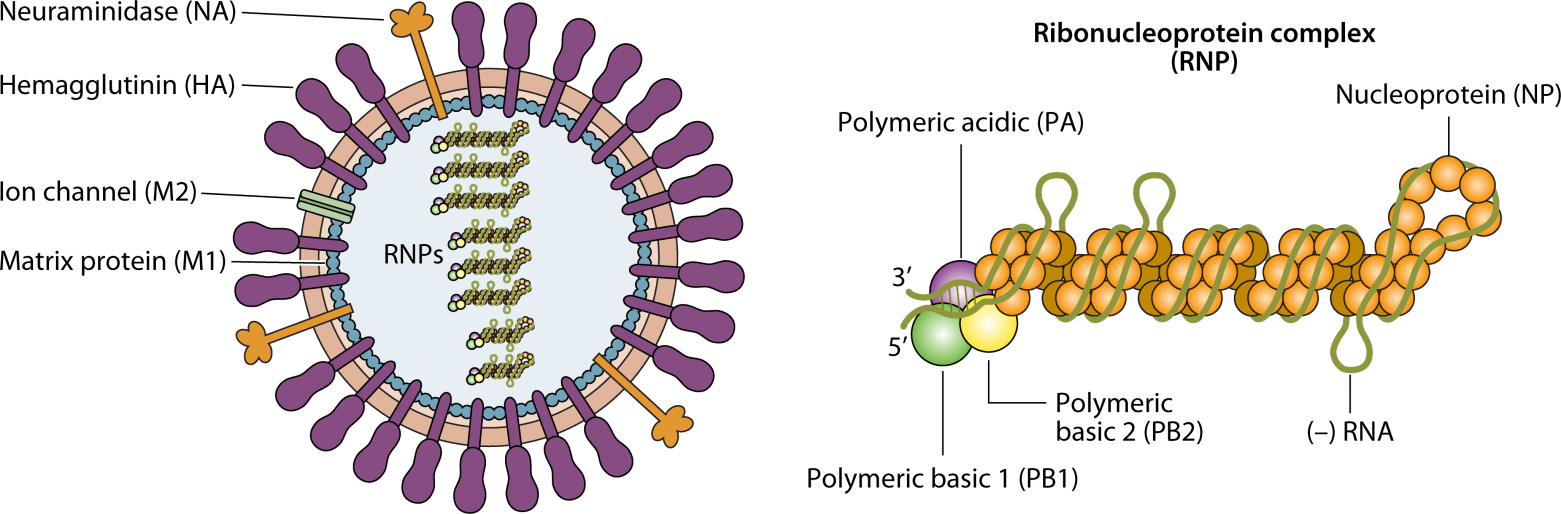
Cartoon representation of influenza virus architecture. The surface of the virion particle is decorated with HA trimers and NA tetramers and M2 ion channels anchored into the lipid bilayer membrane. The M1 protein forms inner matrix layer beneath the envelope. The virion core consists of eight RNP particles (left). RNPs are composed of genomic RNA enwrapped with NP and the heterotrimeric RNA polymerase (RdRp) consisting of PB1, PB2, and PA proteins (right).

Influenza is a segmented negative-sense single-stranded RNA virus that belongs to *Orthomyxoviridae* family (24). IAV genomes contain eight segments that code for 10 essential viral proteins and several strain-dependent accessory proteins (Fig. 1) (25–27). The first three genome segments code for the heterotrimeric RNA-dependent RNA polymerase (RdRp) subunits: the polymerase basic 2 (PB2), polymerase basic 1 (PB1), and the PA proteins (25, 28). The nucleoprotein (NP) is encoded by the fifth genome segment. All of the genomic RNA segments get enwrapped with oligomeric NP and remain associated with a single copy of RdRp to form viral ribonucleoprotein (RNP) complexes that constitute the virus replication machinery (Fig. 1) (25, 29). The fourth and sixth genome segment codes for viral glycoprotein HA and NA that constitute the entry and egress machinery of the virus (30). The seventh genome segment codes for viral matrix proteins M1 and M2. The M2 protein forms the ion channel in the envelope, whereas the M1 protein forms the matrix layer beneath the same and also interacts with the HA, NA, M2, and NP proteins (27, 31, 32). The eighth genome segment codes for non-structural proteins NS1 and NS2. These proteins mainly regulate host immune responses and facilitate viral RNP (vRNP) export from the nucleus, respectively (27). In addition to these viral players, a plethora of host proteins positively or negatively regulate influenza virus propagation (33). In this regard, host kinases play pivotal roles in directly phosphorylating viral proteins to regulate their function or impact various signal transduction pathways thus indirectly impacting virus replication (34). To date, around 28 host kinases are implicated in regulating different steps of the influenza virus life cycle by direct or indirect mechanisms. A proper understanding of this virus-host kinase interplay is essential for creating a comprehensive landscape of influenza virus replication within the host cellular milieu and may open up the avenues of establishing kinase inhibitors as novel anti-influenza drugs, in the future.

**Fig 2:**
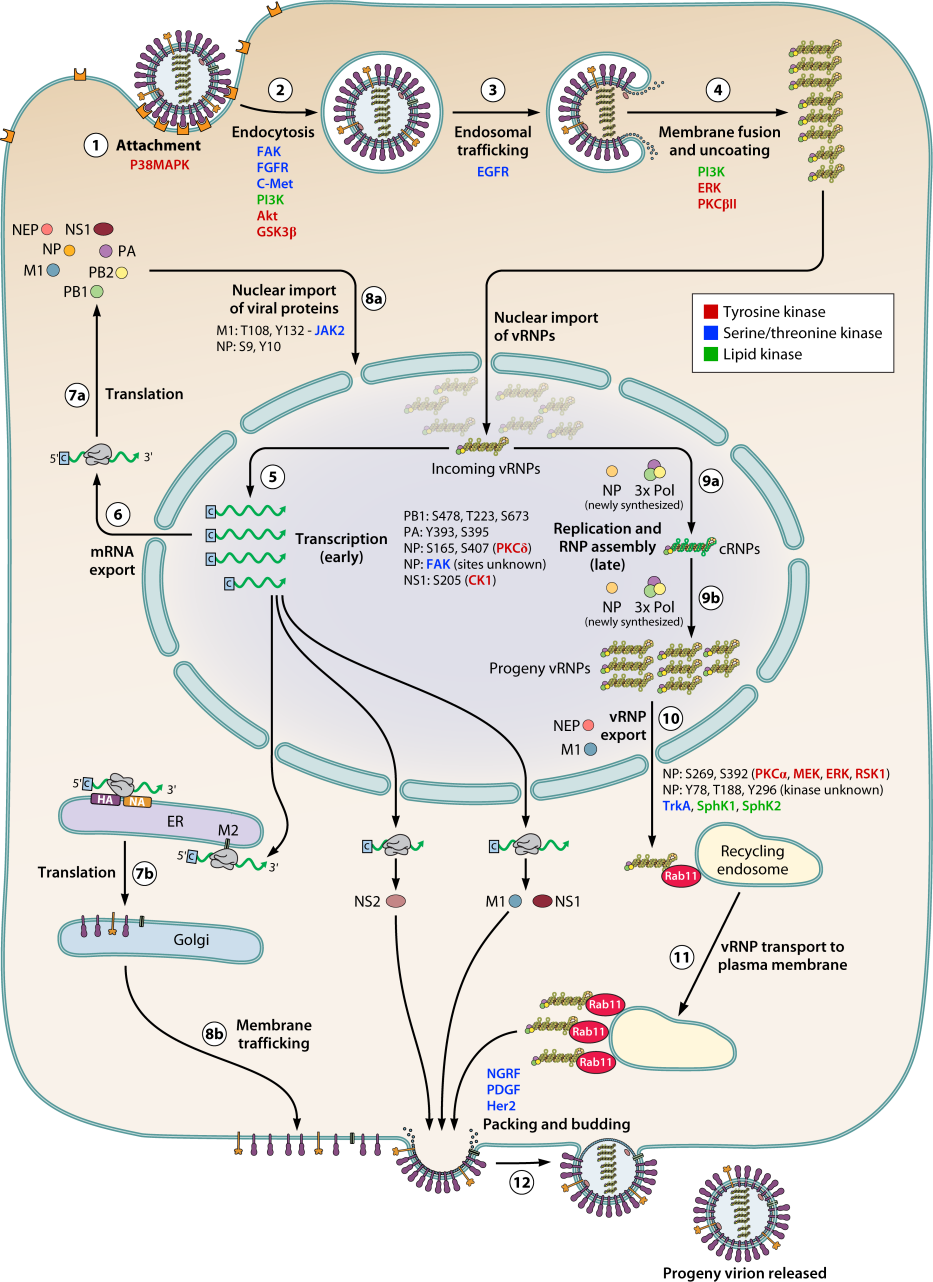
Different phases of influenza virus life cycle and involvement of host kinases. (1) Attachment of virion particle with the host cell surface receptor. (2) Receptor-mediated endocytosis. (3) Endosomes are trafficked through the cytoplasm. (4) Fusion of endosomal membrane and viral envelope releases vRNPs into cytoplasm, which are then transported to the nucleus. (5) Imported vRNPs perform primary transcription to produce viral mRNAs. (6) mRNAs are transported from the nucleus to the cytoplasm. (7a) mRNAs encoding viral polymerase proteins (PB1, PB2, PA, NP, M1, and NEP) get translated into the cytoplasm. (7b) mRNAs encoding viral membrane proteins (HA, NA, and M2) get translated by the endoplasmic reticulum-Golgi network. (8a) Viral non-membranous proteins are transported back to the nucleus. (8b) Membrane proteins are trafficked to the plasma membrane. (9a) vRNPs perform cRNA replication in the presence of newly synthesized proteins thus forming cRNPs. (9b) Positive-sense cRNPs get copied to negative-sense vRNPs. (10) vRNPs are exported out of the nucleus with the help of M1 and NEP proteins. (11) vRNPs are transported to the plasma membrane by Rab11 vesicles. (12) Eight vRNPs are packed into the new virion particle and bud out of the cell by snatching a portion of the host cell plasma membrane already decorated with viral membrane proteins. Host kinases involved in various phases of the life cycle are highlighted in different color schemes as depicted. The entire schematic is oversimplified where the involvement of other host factors is excluded.

In this review, we summarized the role of various host kinases in regulating different steps of the influenza A virus life cycle either by directly phosphorylating viral proteins or indirectly by modulating the host cell environment (Fig. 2; Table 1). By doing so, we have tried to generate a comprehensive overview of how viral proteins and host kinases together orchestrate the replication cycle of the virus. Here, we have discussed about host kinases and corresponding phosphorylation events that have a role in regulating viral replication cycle but consciously excluded the plethora of host kinases and related signaling cascades that are related to antiviral immune response as those constitute a completely independent field of study.

**TABLE 1:** Overview of cellular kinases, their substrates, and their role in the viral life cycle

Phases of life cycle	Nature of the kinase	Name of the kinases	Substrates with phosphorylated amino acids	Role	References
Viral entry	Serine-threonine kinase	P38MAPK	Endosome antigen 1 (T1392) and rabenosyn-5 (S215)	Facilitates endocytic internalization.	Marchant et al. and Macé et al. (35, 36)
		Akt/PKB and GSK3-β	–^*a*^	Promotes viral uptake.	Ehrhardt and Hirata et al. (37, 38)
		ERK	E subunit of the V1 domain of V-ATPase	Ras-activated ERK interacts with the V-ATPase channel of the endosome and decreases interior PH.	Marjuki et al. (39)
		PKCβII	–	Facilitates the release of the virus from the late endosome.	Sieczkarski et al. (40)
	RTK	EGFR, FGFR, and c-Met receptor	PI3K	Transduces signal for internalization process.	Ehrhardt and Eierhoff et al. (37, 41)
	Non-RTK	FAK	–	Promotes actin cytoskeletal reorganization for facilitating endosomal trafficking.	Elbahesh et al. (42)
	Lipid kinase	PI3K	Akt, GSK3-β FAK (Y397) V-ATPase of endosome	Promotes viral uptake through receptor-mediated endocytosis, phosphorylates FAK for facilitating endosomal trafficking, and interacts with the V-ATPase channel of the endosome and acidifies it.	Ehrhardt, Marjuki et al., and Elbahesh et al. (37, 39, 42)
Transcription-replication Dynamics	Unknown	Unknown	PB1 (S478) and PB1 (T223)	Disrupts polymerase function by blocking the NTP entry channel or inhibiting RNA binding.	Dawson et al. (43)
			PB1 (S673)	Suppresses viral transcription.	Dawson et al. (43)
			PA (Y393)	Prevents binding of trimeric polymerase with vRNA and cRNA, thereby impairing polymerase function.	Liu et al. (44)
			PA (S395)	Partially affects mRNA and cRNA synthesis without affecting RNA binding.	Liu et al. (44)
			NP (S9 and Y10)	Regulates NP binding with different isoforms of importins and thereby its nuclear entry.	Zheng et al. (45)
	Serine-threonine kinase	PKCδ	NP (S165 and S407)	Inhibits NP oligomerization and maintains the protein in RNA-free monomeric form.	Mondal et al. (46)
		CK1	NS1 (S205)	Promotes polymerase function by inhibiting DDX21-PB1 interaction.	Patil et al. (47)
	Non-RTK	FAK	NP	Facilitates IAV polymerase activity.	Elbahesh et al. (48)
Nuclear export	Serine-threonine kinase	PKCα, MEK, ERK, and RSK1	NP (S269 and S392)	Facilitates NP-M1 interaction for NP-M1-NEP-CRM1 complex-mediated export of vRNPs.	Schreiber et al. (12)
	Unknown	Unknown	M1 (Y108)	Regulates self-association and nuclear import of M1.	Liu et al. (49)
		Unknown	NP (Y78 and Y296)	Decreases NP-CRM1 interaction and reduces vRNP export.	Zheng et al. and Cui et al. (45, 50)
			NP (T188)	Decreases nuclear export in CRM1-independent way.	Li et al. (51)
	RTK	TrkA	FPPS	CRM1-mediated vRNP export and budding of the mature virion particle.	Kumar et al. (52, 53)
	Non-RTK	Jak2	M1 (Y132)	Regulates nuclear translocation of M1, which in turn regulates nuclear export of vRNPs.	Wang et al. (54)
	Lipid kinase	SphK1 and SphK2	RanBP3	Promotes CRM1- mediated export of vRNPs by inactivating RanBP3	Seo et al. (55)
Assembly, packaging, budding, and release	RTK	NGFR, PDGF, and HER2	FPPS	Facilitates viral particle release.	Kumar et al. (53)

^*a*^ –, absence of a specific phosphorylation site of these kinases.

## THE PHOSPHORYLATION NEXUS REGULATING VIRAL ENTRY

### Triggering viral uptake: EGFR-PI3K-Akt-GSK3 signaling takes the lead

Being obligatory intracellular parasites, viruses have to enter the host cell in order to initiate their replication cycle. For influenza viruses, attachment of viral HA to the host cell surface glycoconjugates marks the initiation of the infection cycle (30, 56). These host glycoconjugates contain terminal sialic acid (SA) moieties that are specifically recognized by the receptor-binding domain of HA (30, 56). Specific binding of HA to the SA receptor, its cleavability by the host proteases, glycosylation pattern, and stability in the low pH environment of the endosomes together determine host specificity and viral tropism (57). In the recent past, two different subtypes of influenza A viruses, H17 and H18, had been isolated from bats, which utilize Major Histocompatibility Complex (MHC) class II protein as an entry receptor instead of the canonical SA moieties (58–60). Irrespective of the receptor specificity, virus attachment to the cell surface induces endocytosis of the virion particle leading to their internalization.

During attachment, high mannose residues on the virus surface glycoprotein get recognized by host cell pattern recognition receptors like Toll-like receptor 4 (TLR4), which activate the p38MAPK in a myeloid differentiation factor 88-dependent manner (35). This is a part of the general host innate immune response that leads to the activation of different transcription factors like nuclear factor kappa B (NF-κB) and induction of proinflammatory cytokines like interleukin-6 and tumor necrosis factor alpha (22). This becomes evident when a specific p38MAPK inhibitor abolishes upregulation of proinflammatory cytokines and protects mice from lethal H5N1 infection (22). Interestingly, the virus is capable of exploiting this antiviral pathway to facilitate its internalization. For example, the TLR4-activated p38MAPK, a serine-threonine kinase, phosphorylates the Rab5 effector proteins like early endosome antigen 1 and rabenosyn-5 at threonine 1392 (T1392) and serine (S215), respectively (36). This in turn facilitates the endocytic uptake of virion particles. SB203580, a specific inhibitor of p38MAPK, inhibits influenza H1N1 virus propagation by blocking viral entry step (35).

Interaction of HA with the SA receptor is not enough to transmit a signal for viral internalization. During viral attachment, IAV induces the formation of clustered lipid rafts that acts as RTKs containing signaling platform. A number of RTKs, namely, epidermal growth factor receptor (EGFR), fibroblast growth factor receptor (FGFR), and c-Met receptors, have been reported to get activated during IAV entry, which presumably transduce signal for the internalization process (41). For example, the role of EGFR-phosphatidyl-inositol-3 kinase (PI3K)-Akt signaling has been investigated in the influenza virus entry process (41). The PI3K, a downstream kinase of EGFR, is activated upon viral attachment. PI3K inhibition has been shown to significantly reduce viral titer by blocking virus internalization into the endosomes (37). It is reported that EGFR promotes IAV uptake through PI3K-dependent receptor-mediated endocytosis (41). Furthermore, Akt, a downstream kinase of the PI3K pathway, is known to regulate viral entry (38). Inhibition of Akt kinase activity by a small peptide called “Akt in” in the H1N1 PR8 virus-infected cells decreases viral uptake and replication without producing inflammatory cytokines (38). “Akt in” treatment suppresses glycogen synthase kinase3-β (GSK3-β) phosphorylation, which is a downstream kinase of Akt, known as a key regulator of influenza virus entry (38).

### FAK controls vesicular trafficking

Influenza viruses are pleomorphic and produce spherical and filamentous virion particles. Internalization of the spherical IAV takes place majorly through clathrin-mediated endocytosis (61), while the filamentous IAVs and some spherical viruses enter the cell through macropinocytosis (62). After uptake, endosomal trafficking occurs toward the nucleus through microtubules and microfilaments. Focal adhesion kinase (FAK), a non-RTK, promotes actin cytoskeletal reorganization that facilitates trafficking of IAV-containing endosomes. FAK gets phosphorylated at tyrosine 397 (Y397) residue upon viral attachment in a PI3K-dependent manner (42). This phosphorylated Y397 residue (FAK-Y397-P) then acts as a binding site for Src kinases, phospholipase C-γ, and PI3K, which in turn mediate maximal FAK activation via phosphorylation of multiple residues, including tyrosine 576/577 (Y576/Y577) (42). FAK inhibition or kinase mutant FAK results in disorganized actin meshwork that leads to the accumulation of IAV endosomes at the cell periphery (42). This in turn blocks subsequent steps of the virus life cycle including the fusion of viral and endosomal membranes and the release of vRNPs into the cytoplasm.

### PKCβII and Ras-ERK/PI3K signaling is indispensable for intracellular invasion of the virus

In the late endosome, acidic pH (pH 5.0) drives the fusion of the endosomal membrane with the viral envelope in an HA-dependent manner. Acidic pH triggers a conformational rearrangement of HA, whereby the fusion peptide in the N-terminal domain of the HA2 subunit gets inserted into the endosomal membrane. The C-terminal transmembrane domain of the HA2 remains embedded in the viral envelope during this event (63). Subsequent structural transition brings the N- and the C-terminal domains of HA2 in close proximity, thereby fusing the viral and host membranes together (27, 63). In a parallel set of events, the low pH environment of the endosome opens up the M2 ion channel in the viral envelope, thereby acidifying the viral interior that disrupts the M1 matrix layer and releases vRNPs into the cytoplasm (64).

Endosomal acidification is tightly regulated through multiple host kinases. Ras, a monomeric GTPase family protein, is known to be activated in the early endosomes after IAV internalization that subsequently activates the PI3Kγ pathway (65). This Ras-mediated PI3K activity was shown to be involved in clathrin-independent endocytosis, endosomal maturation, and intracellular transport of viruses (65). Two downstream kinases of Ras, PI3K, and extracellular signal-regulated kinase (ERK) play a crucial role in acidifying the interior of the late endosome (37, 39). They activate the vacuolar H^+^-ATPase (V-ATPase) channel that decreases endosomal PH and subsequently facilitates membrane fusion. It is reported that activated ERK and PI3K directly interact and co-localize with the E subunit of the V1 domain of V-ATPase (39).

Another serine-threonine kinase, protein kinase C (PKC), influences different phases of the influenza virus life cycle with its 15 cellular isoforms in human (66). One such isoform, PKCβII, is known to facilitate IAV entry (40). A phosphorylation-deficient form of PKCβII significantly inhibits IAV entry by blocking the release of the virus from the late endosomes. PKCβII regulates the sorting of late endosomes, which is an important factor for successful viral entry (40). Interestingly, PKCδ knockout cells also show reduced viral entry that can be rescued by low pH-mediated direct fusion of the virus to the cell membrane (46). Perhaps, multiple PKC isoforms regulate the endocytic entry of influenza viruses through a mechanism that is yet to be fully characterized.

IAVs deliver their vRNPs from the host cell surface to the nucleus within an hour of receptor binding, out of which viral attachment and endocytosis complete approximately within the first 10 minutes. The rest of the time is utilized for uncoating and release of vRNPs from the core of the virion and their transport across the nuclear envelope (67). A series of host factors including kinases are involved in the transport and trafficking of vRNPs across the nuclear membrane, which will be discussed in the subsequent sections.

## HOST KINASE-DEPENDENT DYNAMICS OF VIRAL RNA SYNTHESIS

Newly released vRNPs in the host cell cytoplasm are trafficked to the nucleus through an importin-dependent pathway (68). The first event that occurs within the nucleus is primary transcription that produces viral mRNAs. These mRNAs are then exported out of the nucleus and get translated with the help of the host ribosomes (27). At the later stage, viral genome is replicated through a positive-sense RNA intermediate, the complementary RNA (cRNA), which then serves as the template for the synthesis of the negative-sense viral genome, that is, vRNA. During genome replication, the active RdRp recruits viral NP proteins throughout the length of cRNA/vRNA concomitant to their synthesis, thus leading to the assembly of complementary RNP (cRNP)/vRNP complexes (27, 46).

Influenza viral mRNA transcription is a primer-dependent process, carried out by RdRp, residing within the RNP complex (resident RNA pol) (69). The viral polymerase snatches the 5′ cap of a nascent host transcript and uses it to prime its own mRNA synthesis. The viral polymerase directly interacts with the serine-5 phosphorylated C-terminal tail domain of the host RNA polymerase II, to get access to the 5′ cap structure of the nascent mRNA transcript (70). The cap-binding domain of the PB2 subunit binds to 5′ cap structure of the host nascent mRNA followed by the PA subunit that cleaves the mRNA 10- to 13-nucleotide downstream of the cap using its endonuclease domain. PB2 cap-binding domain then undergoes structural rearrangement, whereby the cleaved capped RNA primer is placed into the catalytic core constituted of the PB1 subunit where this primer is extended using vRNA as a template. After generating a sufficient pool of viral proteins, RdRp shifts into the replication mode (69, 71). During replication, the newly synthesized polymerase associates with the resident RNA polymerase to form a dimer of heterotrimer that executes *de novo* initiation of replication (primer independent), stabilizes the nascent cRNA, and recruits the monomeric NP molecules to the growing RNA chain for efficient RNP assembly. Subsequently, these cRNPs are used as the template for vRNP synthesis (29, 46, 71).

### Phospho-modulation of polymerase function

Host kinases play a critical role in regulating the optimal activity of influenza virus RdRp and switching it between the transcriptase to replicase mode. For example, specific phosphorylation of the PB1 subunit at serine 478 (S478) or threonine 223 (T223) is essential for optimal polymerase function. It has been reported that the introduction of phospho-mimetic mutations at these two sites disrupts polymerase function by blocking the nucleotide triphosphate (NTP) entry channel or inhibiting RNA binding, respectively (43). Another phosphorylation of PB1 at serine 673 (S673) specifically suppresses viral transcription and not replication, thereby maintaining the balance between early transcription and late replication events (43). Interestingly, phospho-ablative mutations at any of these sites significantly attenuate the virus at later time points of post-infection. It is possible that reversible phosphorylation at these residues is important for optimal polymerase function, for transcription-replication balance, and for successful completion of the infectious cycle. Unlike PB1, phosphorylation of the PA subunit at two different residues, tyrosine 393 (Y393) and serine 395 (S395), serves as the negative regulator of polymerase function (44). While constitutive phosphorylation of PA Y393 residue prevents polymerase binding to the 5′-ends of vRNA and cRNA and hence impairs polymerase function, S395 phosphorylation partially affects mRNA and cRNA synthesis without affecting RNA binding. The introduction of phospho-mimetic amino acids at these two positions completely abrogates the assembly of the polymerase and thereby shuts down viral RNA synthesis (44). Interestingly, a phospho-null alanine substitution of the PA phospho-sites does not impact virus replication, which suggests that these phosphorylation events are not essential for polymerase function but rather serve as one of the many antiviral strategies of the host. If that is the case, then it would be worth investigating why these residues were kept conserved by the virus throughout the course of evolution. Nonetheless, it is clear that phosphorylation of different polymerase subunits appears to be critical in regulating different steps of RNA synthesis, although the identity of the host kinases that are involved in these phospho-regulatory events is yet to be unraveled.

Other than the core polymerase proteins, nuclear export protein (NEP) is reported to serve as a co-factor for the H5N1 virus RNA polymerase by interacting with PB1 and PB2 and stabilizing the cRNP complexes (72). NEP is reported to get phosphorylated at several serine residues, although the specific role of these modifications is yet to be elucidated (72).

### Phospho-regulation of NP oligomerization and its nucleocytoplasmic shuttling fine-tune the balance between transcription and replication

**Fig 3:**
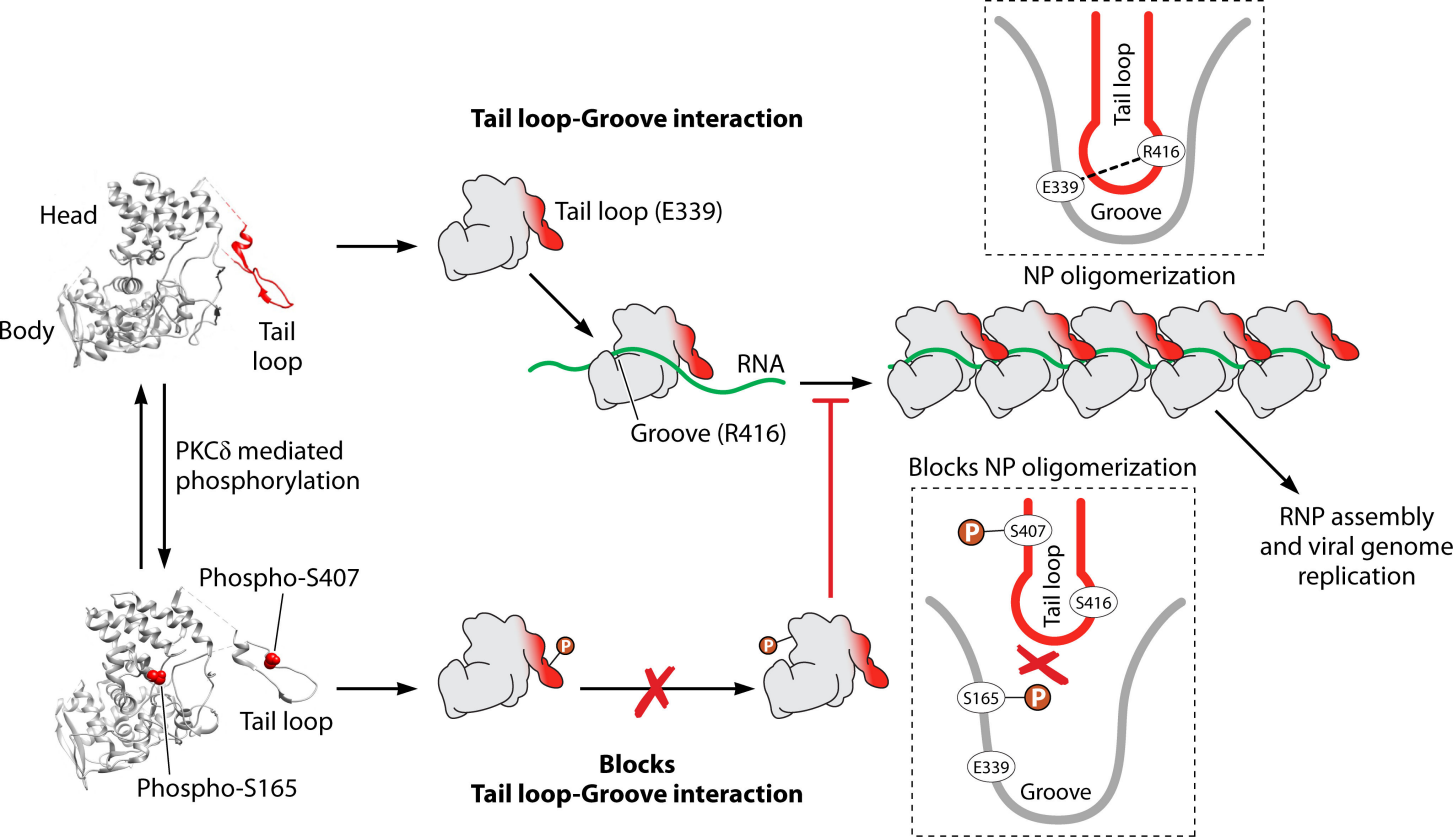
Phospho-regulation of NP oligomerization. Monomeric NP structure (PDB: 2iqh) consists of a head and a body domain and a tail loop extended outward from the core of the protein. Individual NP protomers interact through salt bridge interaction between the tail loop (E339) of one and the groove (R416) of the other. PKCδ-mediated reversible phosphorylation at S407 in the tail loop and S165 in the binding groove sterically inhibits tail loop-groove interaction and thus phospho-modulates NP oligomerization, RNP assembly, and genomic RNA replication. This figure is adapted from Mondal et al. (73).

NP is the major component of the vRNPs and gets extensively phosphorylated at various serine, threonine, and tyrosine residues. NP phosphorylation regulates its nuclear localization and its assembly into the nascent vRNP complexes that ultimately regulate polymerase function and coordinate the balance between transcription and replication (Fig. 3). Newly synthesized NP gets phosphorylated at serine 9 (S9) and tyrosine 10 (Y10) residues that are necessary for its nuclear import (45). Phosphorylation and dephosphorylation at these sites regulate NP binding with different isoforms of importin proteins and thereby its entry into the nucleus, although the responsible kinase remains uncharacterized. Within the nucleus, NP monomers oligomerize along the length of viral genomic/antigenomic RNA concomitant to their synthesis, leading to the assembly of new vRNP/cRNP complexes (73). This is why a steady supply of monomeric NP is a prerequisite for genome replication but is dispensable for primary transcription. Unfortunately, maintaining the pool of monomeric NP is challenging as it binds to cellular RNA non-specifically and oligomerizes to form insoluble aggregates. In this regard, specific phosphorylation at the NP homotypic interface [serine 165 (S165) in the groove and serine 402, serine 403, and serine 407 (S407) in the tail loop] has been shown to inhibit NP oligomerization and maintain the protein in RNA-free monomeric form that acts as the raw material for the RNP assembly process (73). A specific PKC isoform, PKCδ, was reported to phosphorylate NP at both S165 in the binding groove and S407 at the tail loop region and thereby blocks the “tail loop-groove interaction,” which is critical for NP:NP interaction (46). Furthermore, active PKCδ interacts with the PB2 subunit of RdRp during the course of infection, possibly mediating specific recruitment of monomeric NP to the nascent RNA chain coming out of the active core of the polymerase. Thus, without affecting the primary transcription, host PKCδ phospho-regulates vRNP assembly, required for the transition of the polymerase from transcriptase to replicase mode (46).

NP has also been reported to be closely associated with FAK, which plays a critical role in viral transcription and replication independent of its role in viral entry (48). FAK facilitates IAV polymerase activity, and the introduction of a FAK inhibitor or overexpression of the kinase-dead mutant restricts viral RNA synthesis in the context of reconstituted viral polymerase activity assay. During infection, FAK inhibition significantly reduces the synthesis of viral RNAs, mRNA, vRNA, and cRNA levels by 500-folds at 8 Hours Post Infection (hpi) (48). Interestingly, the kinase-dead mutant of FAK still retains its ability to interact with NP, suggesting that both the kinase and the scaffolding activity are essential for its pro-viral role toward the influenza virus (48). Nonetheless, it is not clear whether FAK phosphorylates NP or any of the polymerase subunits to execute its function upon viral RNA synthesis.

Viral NS1 protein, which acts to antagonize the host antiviral response, has also been shown to promote polymerase function although the detailed mechanism is yet to be characterized. NS1 is known to get phosphorylated at serine 205 (S205) position by the cellular serine-threonine kinase, the casein kinase 1 (CK1) (47). This CK1-mediated phosphorylation enables NS1 to interact with cellular DEAD box protein DDX21. DDX21 is reported to inhibit viral RNA synthesis by binding to PB1 and inhibiting the formation of the heterotrimeric polymerase complex (74). It has been proposed that phosphorylated NS1 binds DDX21 and sequesters it from the polymerase complex, thereby antagonizing its antiviral role and promoting viral polymerase activity.

## DIVERSE HOST KINASES ORCHESTRATE NUCLEAR EXPORT OF VRNPS: A MULTIFACETED REGULATORY NETWORK

**Fig 4:**
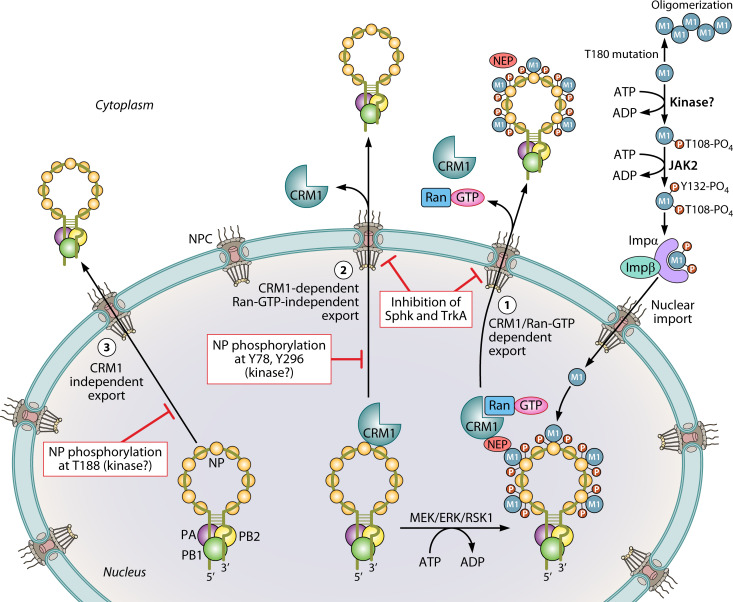
Mechanism of vRNP export and involvement of host kinases. Y132 and T108 phosphorylation regulates nuclear entry of M1 protein. MEK-ERK-RSK1-mediated phosphorylation at S269 and S392 enables NP to interact with M1 and thereby facilitates Crm1-Ran-GTP-dependent export of vRNPs through the intermediacy of NEP. However, NEP interacts with viral polymerase for facilitating this way of transport, not shown here. Specific NP phosphorylation at Y78 and Y296 inhibits its interaction with Crm1, thereby inhibiting Crm1-dependent Ran-GTP-independent export of vRNPs. TrkA and Sphk promote Crm1-mediated export of vRNPs. Crm1-independent export of vRNPs is also inhibited by specific NP phosphorylation at T188 by unknown kinases.

After synthesis, progeny vRNPs are actively exported out of the nucleus and transported to the plasma membrane to assemble into mature virion particles that then bud out. Viral proteins NP, M1, and NEP in conjunction with the host chromosome region maintenance protein 1 (CRM1) execute the vRNP transport across the nuclear pore complex (Fig. 4) (75). This also requires the participation of the monomeric GTPase family of proteins like Ran and Rab, especially Rab11 (76). CRM1 is an exportin that binds with the nuclear export signal (NES) of the target proteins and mediates their export from the nucleus. Viral M1 protein contains NES that interacts with CRM1 through the intermediacy of viral NEP protein (77, 78). NEP through its C-terminus binds with M1 while its N-terminal NES interacts with CRM1. The NEP-bound M1 also interacts with NP within the vRNP complexes. It ultimately leads to the formation of vRNP-M1-NEP-CRM1 complex and nuclear export of vRNPs (Fig. 4) (75), although alternatively proposed daisy chain model of vRNP export says that NEP interacts with the viral polymerase also to provide an additional binding site and support to M1-vRNP binding (not shown in Fig. 4) (79). NP alone contains three NES (NES1, 2, and 3) that facilitate the nuclear export of vRNPs (80). Out of the three, only NES3 interacts with CRM1, but this interaction is not Ran-GTP dependent (81). NES1 and NES2 can facilitate vRNP export without the involvement of CRM1. Nuclear retention of NP was observed when all three NES were mutated simultaneously (80).

### Unlocking the gate through phosphorylation of M1 and NP

Nuclear export of vRNPs should be tightly regulated in order to ensure their cytoplasmic localization only at the later stage of the virus life cycle. Phosphorylation of viral proteins plays a critical role in these dynamics of vRNP export (Fig. 4). For example, phosphorylation of M1 at tyrosine 132 (Y132) regulates its nuclear translocation, which in turn is essential for the nuclear export of vRNPs (54). Mutation of M1 at Y132 in A/WSN/1933 (H1N1) virus alters its interaction with importin α and restricts its nuclear entry, thereby reducing vRNP export and virus titer. AG490, a JAK1 and JAK2 inhibitor, stops M1 from entering the cell’s nucleus when applied to A549 cells lacking JAK1 kinase activity. These suggest that JAK2, a non-RTK, is responsible for phosphorylating M1 at Y132, thereby modulating its nuclear translocation (54). Other than Y132, M1 is reported to be phosphorylated at threonine 108 (T108) that regulates its multimerization. Mutation of T108 to a non-phosphorylatable alanine strongly increases its self-association at the cell membrane, which in turn decreases its nuclear import, hence reducing vRNP export (49). The specific kinase behind this T108 phosphorylation is still unknown. Apart from M1, phosphorylation of NP also regulates vRNP export. Phosphorylation at tyrosine 78 (Y78) inhibits NP binding to CRM1, thereby delaying vRNP nuclear export and ultimately restricting virus growth. Janus and Src kinases are suggested to phosphorylate NP at this Y78 residue (50). Another phosphorylation on tyrosine 296 (Y296), which is closely located to NES3 of NP, reduces its interaction with CRM1, thereby increasing its nuclear retention and impacting the progression of the infectious cycle (45). Phosphorylation of NP at threonine 188 (T188), located within the NES2, hampers NES2-dependent yet CRM1-independent nuclear export of NP (51), although the specific kinases behind this Y296 and T188 phosphorylation are still unknown. In addition to M1 and NP, the NEP phosphorylation at its highly conserved serine 23–25 (S23–25) residues was reported to facilitate vRNP export (82), although the responsible kinases remain elusive.

### Mastering vRNP nuclear export: insights into the role of the Raf/MEK/ERK/RSK Cascade and TrkA signaling

The Ras/Raf/MEK/ERK cascade constitutes one of the most important signaling pathways regulating the nuclear export of influenza vRNPs (83). Later in the replication cycle, viral HA proteins accumulate into the lipid rafts of the host cell membrane, which activates the Raf/MEK/ERK pathway through the activation of PKCα; this, in turn, promotes vRNP export (84). RSK1, a serine-threonine kinase, is one of the downstream effectors of the Raf/MEK/ERK pathway that was reported to be the key player for the vRNP export process. RSK1 phosphorylates NP at serine 269 (S269) and serine 392 (S392), which is essential for its binding to M1 and thus for the formation of NP-M1-NEP-CRM1 complex (Fig. 4) (12). Inhibition of either MEK or RSK decreases M1 binding and leads to nuclear retention of vRNPs. RSK has two subtypes, RSK1 and RSK2. In contrast to the pro-viral role of RSK1, RSK2 acts as an antiviral factor by activating NF-κB and interferon beta (IFN-β) pathways (85). It is interesting that two downstream effectors (RSK1 and RSK2) of the same pathway (Raf/MEK/ERK) exert opposite effects in the context of the influenza virus propagation.

The nerve growth factor receptor (NGFR) TrkA, an RTK, plays a crucial role in influenza A virus replication. TrkA knockdown cells support lower levels of viral multiplication, and its inhibition with a specific inhibitor (AG879) reduces post-entry steps including (i) synthesis of viral RNA (mRNA, vRNA, and cRNA), (ii) CRM1-mediated nuclear export, and (iii) budding of mature virion particles from the plasma membrane (52, 53). Further studies are required to elucidate the precise mechanism by which TrkA inhibition shows such a multifaceted impact on influenza A virus life cycle. Sphingosine kinases (SphK1 and SphK2) are a group of lipid kinases that convert sphingosine to sphingosine-1-phosphate and are known to modulate NF-κB, PI3K/Akt/mTOR, and Ras/Raf/MEK/ERK pathway (86). SphK1 acts as a pro-viral factor during influenza A virus infection. Blocking of SphK interferes with CRM1-mediated nuclear export of vRNPs by inactivating Ran-binding protein 3 (RanBP3), a co-factor of CRM1. SphK inhibition also leads to altered phosphorylation of Akt, p^90^RSK, and ERK, which in turn modulates CRM1-RanBP3 interaction (55). Clearly, multiple host kinase cascades converge at the CRM1-mediated nuclear export of vRNPs although the precise connection between these cascades is yet to be established.

## THE FINAL ACT: DYNAMIC INTERPLAY OF ASSEMBLY, PACKAGING, BUDDING, AND RELEASE IN VIRAL EXIT

IAV envelope is derived from the host cell membrane “lipid raft” region, enriched with cholesterol and sphingolipids (87). To generate an infectious virion, one copy of each of the eight vRNPs must be packed within the matrix layer followed by the envelope. There must be some specific mechanisms that assemble eight vRNPs into the raft region of the host cell membrane that is already decorated with HA, NA, M2, and M1. Alternatively, it is possible that HA and NA first create a membrane microdomain with a unique lipid composition that attracts M1 and there by eight vRNPs (27). Subsequently, a membrane curvature is introduced by molecular crowding on one leaflet of the bilayer, by the accumulation of curved or bending proteins within the bilayer, or by cytoskeletal remodeling. This is further modulated by membrane-embedded HA, NA, and M2 proteins (27, 88). The final release of IAV bud is completely dependent on the sialidase activity of NA (89). Upon budding, progeny virions remain attached to the host cell membrane through the viral HA binding to the SA moieties. NA hydrolyzes the glycosidic linkage that attaches the SA to the underlying sugar molecule thus disrupting the HA-SA linkages at the cell surface. After release, the movement of viruses in the respiratory epithelium is difficult due to the presence of a protective mucus layer. It is reported that NA-mediated cleavage of SAs from mucins facilitates IAV movement in the respiratory epithelium and increases infectivity (27, 89).

Phosphorylation of viral M1 proteins has been shown to regulate virion assembly process. Recombinant viruses carrying phospho-null mutation at M1 Y132 (Y132A) showed a defect in its lipid raft localization due to reduced interaction with viral HA. This resulted in the diminished structural stability of progeny virions and the formation of filamentous particles (90). Farnesyl diphosphate synthase (FPPS), an enzyme essential for isoprenoid biosynthesis, is reported to affect membrane fluidity and lipid raft formation during IAV release and thus modulate virion budding (91). TrkA, in addition to its role in CRM1-dependent nuclear export of vRNPs, also promotes virion budding in FPPS-dependent manner. TrkA inhibition is reported to restrict FPPS activation and thereby reduce virion budding (53, 91). In addition to TrkA, RTKs also influence virus release. It is reported that two RTK inhibitors that specifically inhibit NGFR, human growth factor receptor 2 (HER2), and platelet-derived growth factor (PDGF) can target FPPS to inhibit viral particle release (53). However, how RTKs regulate FPPS function remains unknown.

## THE QUEST FOR COMPLETENESS: ADDRESSING THE MISSING LINKS

Although a large number of host kinases and their corresponding phosphorylation sites on viral proteins have been mapped, the list is far from complete. Hutchinson et al. (92) performed a phosphoproteome analysis of influenza A and B viruses and identified a large number of putative phosphorylation sites on different viral proteins. A number of these phosphorylation sites are yet to be characterized in terms of their precise role in the virus life cycle (Table 2). For example, the HA gets phosphorylated at threonine 358 (T358), and NA harbors phosphorylation sites at serine 160, 164, and 166 (S160/S164/S166). Neither the role of these phosphorylation events in the virus life cycle nor the kinases responsible for these phosphorylation events are elucidated to date. Similarly, the NP protein is known to get phosphorylated at serine 377 (S377), threonine 378 (T378), serine 457 (S457), serine 472 (S472), and serine 473 (S473) residues. However, the role of these phosphorylation events in the virus life cycle and the responsible kinases are not characterized yet. It is interesting that the NP S473 is least conserved, with the phospho-acceptor serine residue observed only in the case of a few H1N1 and H3N2 strains, thus indicating a strain-specific role of this phosphorylation. Viral NS1 protein was reported to get phosphorylated at threonine 215 (T215) by a subset of cellular proline-directed kinases including cyclin-dependent kinases (CDKs) and ERKs (93). Alanine substitution of T215 results in reduced viral propagation at early time points of post-infection, although the precise mechanism remains elusive (93). Other than T215, S42, and S48 were also reported to get phosphorylated in recombinant influenza A/Udorn/72 (H3N2) virus NS1 protein. PKCα-mediated phosphorylation of NS1 at S42 has been shown to regulate its double-stranded RNA binding ability and hence virus replicability (94). The S42 phosphorylation of NS1 was not detected in 2009 H1N1 indicating a strain-specific role of this phosphorylation (94).

**TABLE 2:** Phosphorylation sites of different viral proteins with unknown kinases or unknown physiological roles or both unknown

Viral proteins	Phosphorylation sites	Kinases	Role	References
PB1	S478 and T223	Unknown	Disrupts polymerase function by blocking the NTP entry channel or inhibiting RNA binding.	Dawson et al. (43)
	S673	Unknown	Suppresses viral transcription.	Dawson et al. (43)
PB2	S472	Unknown	Unknown	Hutchinson et al. (92)
PA	Y393	Unknown	Prevents binding of trimeric polymerase with vRNA and cRNA, thereby impairing polymerase function.	Liu et al. (44)
	S395	Unknown	Partially affects mRNA and cRNA synthesis without affecting RNA binding.	Liu et al. (44)
	S224/S225	Unknown	Unknown	Hutchinson et al. (92)
HA	T358	Unknown	Unknown	Hutchinson et al. (92)
NP	S9 and Y10	Unknown	Regulates NP binding with different isoforms of importins and thereby its nuclear entry.	Zheng et al. (45)
	Y78 and Y296	Unknown	Decreases NP-CRM1 interaction and reduces vRNP export.	Zheng et al. and Cui et al. (45, 50)
	T188	Unknown	Decreases nuclear export in CRM1-independent way.	Li et al. (51)
	S377, S472, S473, S450, and S457 and T378	Unknown	Unknown	Hutchinson et al. (92)
NA	Serine 160, 164, and 166	Unknown	Unknown	Hutchinson et al. (92)
M1	T108	Unknown	Regulates self-association and nuclear import of M1.	Liu et al. and Hutchinson et al. (49, 92)
	S2, S195/S196, and S224/S225/S226, T5, T9, T37, and T168/T169, and Y10	Unknown	Unknown	Hutchinson et al. (92)
M2	S64 and T65	Unknown	Unknown	Hutchinson et al. (92)
NS1	T215	CDKs and ERKs	Unknown	Hsiang et al. (94)
	S42	PKCα	Unknown	Hsiang et al. (94)
	S48 and T197	Unknown	Unknown	Hutchinson et al. and Hsiang et al. (92, 94)
NEP	S25	Unknown	Facilitates nuclear export.	Hutchinson et al. and Reuther et al. (92, 95)

The area that largely remains unexplored is the elucidation of the role of host kinases in determining viral tropism, pathogenesis, and transmission. Unfortunately, majority of the kinase-related studies were done in cancerous human lung epithelial cells (A549) lacking validation of the associated phenotypes in primary airway epithelial cultures or in animal models. Tissue- or cell-specific expression of kinases (96, 97) may control their susceptibility or permissiveness toward infection and thus determine species-specific replication fitness of influenza virus. In this regard, it has been shown that preferential activation of p38MAPK in human airway epithelial cells during influenza virus infection makes them more susceptible toward influenza virus than a respiratory syncytial virus (35). Studies investigating host-specific phosphorylation of influenza virus are spars, although strain- and subtype-specific phosphorylation events are documented (98). Recently, a functionally important phosphor-acceptor site has been identified in PB2, replacing which with phospho-mimicking glutamic acids boosts polymerase function. The PB2-282nd residue is serine in the case of bat influenza virus (H17N10 and H18N11) polymerase, whereas the corresponding position harbors a conserved glutamic acid in the case of classical influenza A, B, C, and D viruses (99). It is an open question whether the PB2-S282 residue gets phosphorylated in the case of bat influenza virus and whether such host-specific phosphorylation of PB2 in bats helps the unusual bat influenza viruses to achieve higher replicability in their native host species. Further investigations are required to elucidate such species-specific phosphorylation and associated host kinases that may have shaped up influenza virus evolution in a wide variety of host species.

To conclude, there are still immense knowledge gaps in terms of unraveling the identity and intricacies of host kinases that orchestrate the viral life cycle. Shedding light upon these intricacies holds the key to unveiling the enigmatic interplay between viruses and the host’s kinome. As the kinases are involved in a multitude of cellular processes and signaling networks, determining their specific role in virus replication remains technically challenging. The introduction of newer techniques like CRISPR-based inducible knockouts (82, 100, 101) and chemical degron systems (102–104) can help in overcoming these long-standing challenges. In fact, several kinases within PKC and MAPK family have been listed as pro-viral host factors identified in genome-wide CRISPR screening studies (101). Future screens targeted toward host kinome may help in gaining deeper understanding of this complex virus-host interplay that may provide novel insights and pave the way for the development of potential antiviral drugs in the form of kinase inhibitors.
